# Current dependence of the negative magnetoresistance in superconducting NbN nanowires

**DOI:** 10.1038/s41598-022-26475-6

**Published:** 2022-12-20

**Authors:** Zoharchen Sofer, Avner Shaulov, Yosef Yeshurun

**Affiliations:** Institute of Superconductivity and Institute of Nanotechnology, Department of Physics, Bar-Ilan University, 5290002 Ramat-Gan, Israel

**Keywords:** Nanoscale devices, Nanoscale materials, Other nanotechnology, Magnetic properties and materials, Surfaces, interfaces and thin films, Nanoscience and technology, Physics, Condensed-matter physics, Magnetic properties and materials, Superconducting properties and materials, Surfaces, interfaces and thin films

## Abstract

Magnetoresistance measurements in amorphous NbN nanowires show that transport current affects their negative magnetoresistance (nMR) in a manner qualitatively similar to temperature. In particular, the current suppresses the nMR and, beyond a certain level it eliminates the effect altogether. As the temperature dependence of the nMR effect is more pronounced at low currents, similarly the current dependence of the effect is more pronounced at low temperatures. These results are discussed in terms of the phenomenological model which attributes the nMR to the interplay between the resistance originating from the rate of phase slips via the Josephson relation and the Ohmic contribution from quasiparticles charge imbalance that accompany fluctuations of the order parameter in the nanowire.

## Introduction

Extensive studies have shown that thermal and quantum fluctuations in superconducting nanowires give rise to a non-vanishing resistance below the transition point, down to very low temperatures^1–6^. Each fluctuation is associated with a phase slip of $2\pi ,$ thus inducing voltage according to the Josephson relation $V=\frac{h}{2e}\frac{\partial \varphi }{\partial t}$. Fluctuations have been also considered as the origin of the negative magnetoresistance (nMR) effect^7–9^ found in a wide variety of 1D superconductors^7,10–21^. A phenomenological model by Arutyunov^8^ explains the nMR as originating from a competition between two mechanisms: thermodynamic fluctuations of the order parameter and quasiparticles (qp) charge imbalance which accompanies each phase slip event. The first process provides the conventional positive magnetoresistance, while the second one gives negative contribution as the quasiparticle charge imbalance length decreases with field. In the range of low magnetic fields, the first process is not significantly affected, while the second one is effectively suppressed, contributing to the experimentally observed nMR. Apparently, both processes depend not only on the magnetic field but also on temperature and the transport current. Yet, while most of the published experimental results describe the temperature dependence of the nMR measured at a certain constant current^7,10,12,14–16,21^ data on the effect of the current on the nMR is scarce^17^. In the present study we experimentally explore the influence of transport current on the nMR in amorphous quasi-1D NbN wires. We observe that the current suppresses the nMR effect and, above a certain level it eliminates the effect completely. As the temperature affects the nMR in a qualitatively similar way, the current dependence of the nMR effect is more pronounced at low temperatures. These results are discussed in the framework of the Arutyunov's phenomenological model^8^.

## Experimental

**Figure 1:**
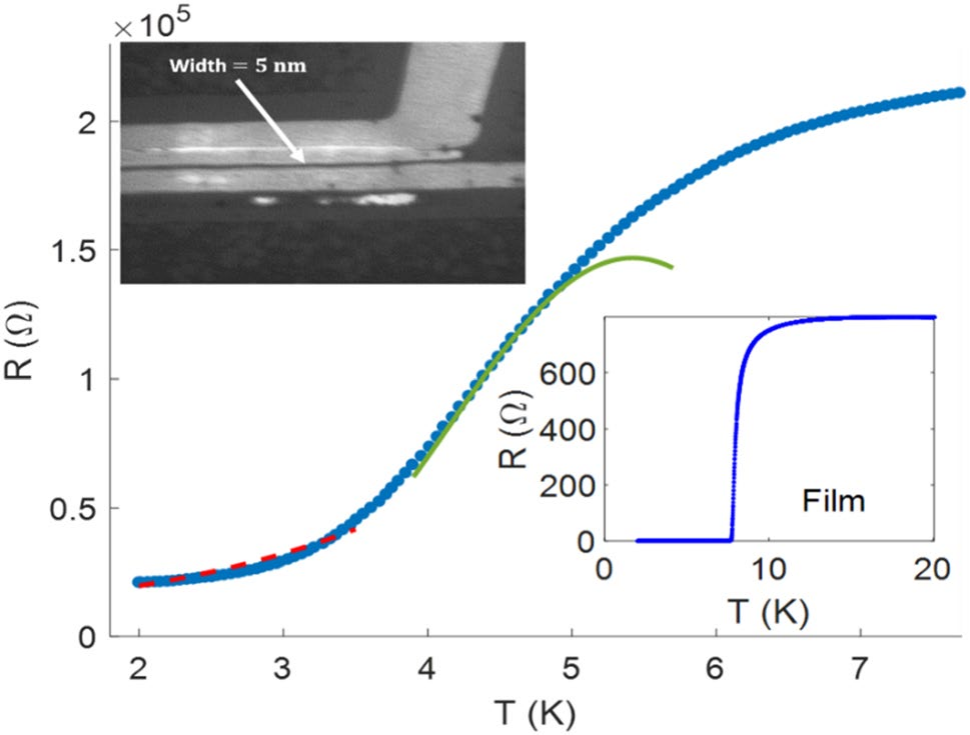
Resistance vs. temperature for the NbN nanowire (main panel) and the film (right inset). The solid and dashed lines in the main panel are fits to theoretical predictions for TAPS (Eq. (1)) and QPS (Eq. (2)), respectively. The wire parameters are ${R}_{n}=2.1*{10}^{5}\left(\Omega \right), width=5 \, \left(\text{nm}\right), \, thickness=5 \,  \left(\text{nm}\right), \, resistivity=87.5 \left(\frac{\upmu \Omega }{m}\right)and \,  {T}_{c}\left(at \, 0.9{R}_{n}\right)=6.5 \,  \left(K\right).$ Left inset shows HR-SEM image of the 5 nm wire.

5 nm films of NbN were deposited on a $1x1 c{m}^{2}$ R-plane sapphire substrate, 500 $\mu m$ thick, using AJA DC reactive magnetron sputtering. For achieving high-quality films, the substrate was first heated to $800^\circ{\rm C}$ for two hours and then cooled to 750 $^\circ{\rm C}$ at which the sputtering took place^22,23^. The sputtering was done at a rate of 0.075 Å per second with a 99.95% pure Nb target in a gas mixture of Nitrogen (8%) and Argon with a total pressure of $2 mTorr$. XRD measurements confirmed the amorphous nature of the films. The inset to Fig. 1 shows the temperature dependence of the resistance of the NbN film, indicating a transition temperature of ~ 7.9 K. The NbN film was patterned into ~ 5 nm wide wires, using Crestec CABL-9510CC High Resolution Electron Beam Lithography and reactive ion etching (RIE), employing $C{l}_{2}-BC{l}_{3}$ process for 50 s. The wire is created by using positive PMMA and exposing two separate lines by electron beam lithography; the wire width is defined as the distance between the two lines. The transport properties of the wires were measured using 4-probes with a distance of $6 \mu m$ between the voltage probes. The magneto-transport measurements were done using Quantum Design physical properties measurement system (PPMS), applying the field in a direction perpendicular to the sample.

## Results

Figure 1 shows the resistance of a NbN nanowire at zero field, exhibiting a broad transition into a superconducting state with a non-vanishing resistance down to 1.8 K. This behavior indicates effects of thermally activated (TAPS) and quantum phase slips (QPS) in this quasi-1D wire, as its lateral dimensions (~ 5 × 5 nm^2^) are of the order of the zero-temperature coherence length in bulk NbN (ξ_0_ = 6 nm).

**Figure 2:**
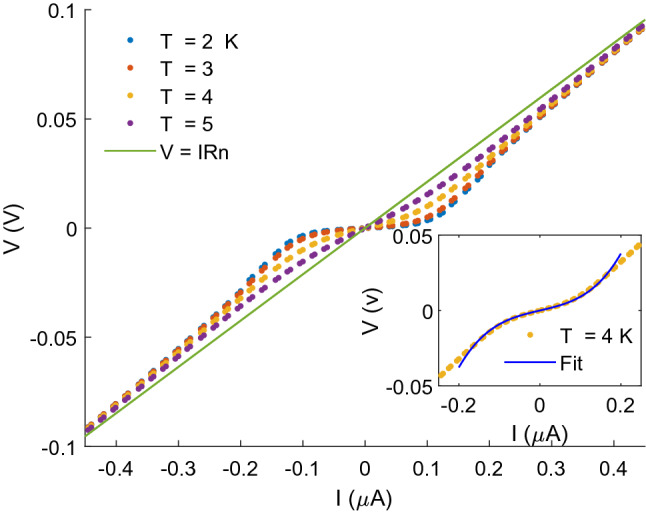
Voltage vs. current for the NbN nanowire at the indicated temperatures. The solid line indicates the Ohmic behavior above T_c_. Inset: Fit of Eq. (1) to the data at 4 K.

**Figure 3:**
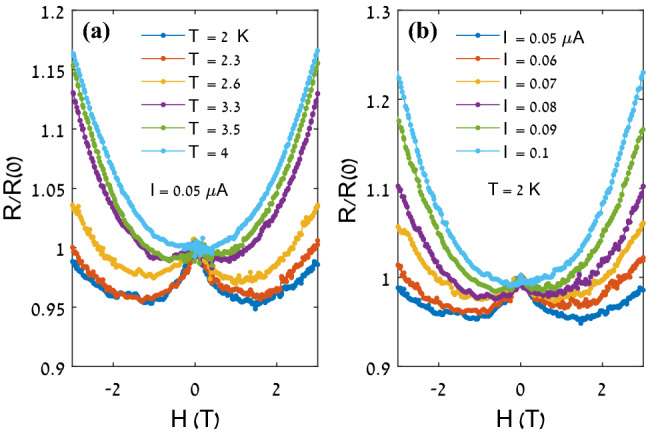
Magnetoresistance behavior of the NbN nanowire, at different temperatures (**a**) and different transport currents (**b**).

Figure 2 shows V-I curves of the NbN nanowire at different temperatures. The curves exhibit a nonlinear increase of the voltage with the current, converging into a linear increase (solid line in the figure) with a slope corresponding to the normal resistance above T_c_. Figure 3a,b show the magnetoresistance behavior at different temperatures and different transport currents, respectively, normalized to the resistance at zero field. The temperature was varied between 2 and 4 K at a constant current of 0.05 μA, and the current was varied between 0.05 and 0.1 μA at a constant temperature of 2 K. Notably, the temperature dependence and the current dependence of the nMR effect are qualitatively similar. An increase in either temperature or current suppresses the effect, and the effect totally disappears at temperatures above 4.5 K or current above 0.1 μA.

**Figure 4:**
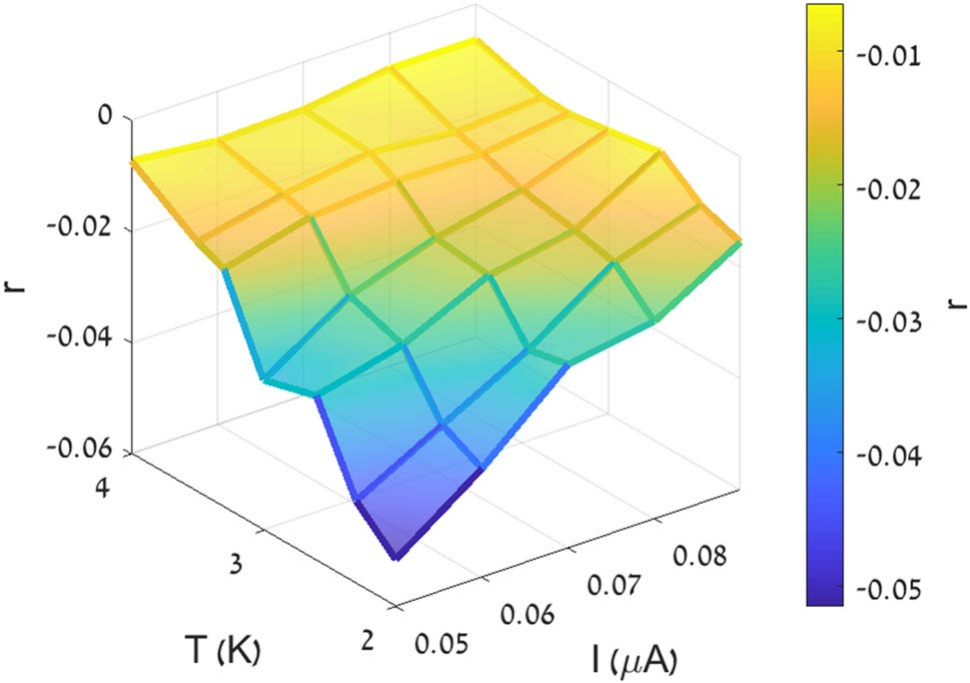
Size of the nMR effect, r, measured as a function of temperature and current.

A more comprehensive description of the dependence of the size of the nMR effect on temperature and current, is presented in the 3-dimensional plot shown in Fig. 4. The size of the effect is defined by the parameter r:

$$r=\frac{R\left({H}_{min}\right)-R\left(0\right)}{R(0)}$$

where $R\left(0\right)$ and $R\left({H}_{min}\right)$ are the resistance at zero magnetic field and at a field ${H}_{min}$ where the magnetoresistance reaches its minimum value. Evidently, the magnitude of the nMR effect decreases monotonically with both temperature and current. The temperature dependence of the effect is more pronounced at low currents. Similarly, the current dependence of the effect is more pronounced at low temperatures.

## Discussion

To verify the fluctuation origin of the broadening transition, we fitted the data of Fig. 1 to the theoretical predictions of Langer, Ambegaokar, McCumber and Halperin (LAMH)^1,2^, taking into account the effect of both temperature and current. According to this theory, the time average voltage generated by thermally activated phase slips (TAPS) is given by^4,5,24^:

1 $${V}_{TAPS}\left(T\right)\cong 2{\Phi }_{0}\Omega \left(T\right)\mathrm{exp}\left(-\frac{\Delta {F}_{0}}{{k}_{B}T}-{\left(\frac{2}{3}\right)}^\frac{1}{2}\frac{{I}^{2}}{3\pi {I}_{1}{I}_{c}}\right)\mathrm{sinh}\left(\frac{I}{2{I}_{1}}\right)$$

where $\Omega =\frac{L}{\xi \left(T\right){\tau }_{GL}}{\left(\frac{\Delta {F}_{0}}{{k}_{B}T}\right)}^\frac{1}{2}$ is the attempt frequency, $\Delta {F}_{0}=\frac{8\sqrt{2}}{3}\frac{{B}_{c}^{2}\left(T\right)}{2{\mu }_{0}}A\xi \left(T\right)$ is the energy barrier for phase slips (proportional to the superconducting condensation energy), ${I}_{1}={k}_{B}T/{\Phi }_{0}$ is the characteristic current above which most phase slippages go in the driven direction and ${I}_{c}=\frac{{\left(\frac{2}{3}\right)}^\frac{1}{2}\pi \Delta {F}_{0 }}{{\Phi }_{0}}$ is the mean-field critical current,$A$ is the wire cross-section, $\xi \left(0\right)$ is the coherence length, ${B}_{c}$ is the thermodynamic critical field, ${\Phi }_{0}$ is the flux quantum and ${\tau }_{GL}$=10^–10^ s. The temperature dependence of $\Delta {F}_{0}$ can be derived assuming ${B}_{c}\left(T\right)={B}_{c}(0)(1-\frac{T}{{T}_{c}})$ and $\xi \left(T\right)= \xi \left(0\right){\left(1-\frac{T}{{T}_{c}}\right)}^{-\frac{1}{2}}$, yielding $\Delta {F}_{0}={E}_{0}{(1-\frac{T}{{T}_{c}})}^{3/2}$ , where ${E}_{0}=\frac{8\sqrt{2}}{3}\frac{{B}_{c}^{2}\left(0\right)}{2{\mu }_{0}}A\xi \left(0\right).$ Attempting to fit Eq. (1) to the R(T) data in the transition region, yields a reasonable fit (solid line in Fig. 1), for ${E}_{0}=5.4x{10}^{-22} J$ and ${T}_{c} = 7.7 \,\text{K}.$ Using the measured value of $A=25 n{m}^{2}$ and the reported values of ${B}_{c}\left(0\right)$= 0.2 T and ξ_0_ = 4 nm for NbN films^25,26^, one obtains for E_0_ a value which is an order of magnitude higher, suggesting that the experimental value for the energy barrier is lower than the theoretically predicted one. We note that similar deviations were reported in previous publications, see e.g. Ref^27^, calling for further investigations. As suggested in that reference, the deviation may be associated with a smaller effective value of A. We also note that the critical field for a film is larger than B_c_(0) of the bulk by a factor of λ/d; our film is 5 nm thick whereas the London penetration depth is ~ 150 nm.

Using the *same* parameters, we fit Eq. (1) to the measured V-I curves of Fig. 2. The inset to the Figure shows such a fit to the V-I curve measured at T = 4 K. Apparently, a good fit is obtained for currents up to I_c_ $\approx 0.2 \,  \upmu A$ for this temperature.

The 'tail' at low temperatures, below $3.8 K$, can be related to quantum phase slips (QPS)^3^. The resulting resistance is expressed in terms of the action ${S}_{QPS}={B}_{2}\left(\frac{{R}_{q}}{{R}_{n}}\right) \left(\frac{L}{\xi \left(T\right)}\right)$^28–31^:

2 $${R}_{QPS}\left(T\right)={B}_{1}{R}_{q}{S}_{QPS}\left(\frac{L}{\xi \left(T\right)}\right)\mathrm{exp}\left(- {S}_{QPS}\right)$$

where ${B}_{1}$ and B_2_ are constants, ${R}_{q}=\frac{h}{4{e}^{2}}$ is the quantum resistance, $L = 6 \mu m$ is the wire length, $\xi \left(T\right)=\xi \left(0\right) {\left(1-T/{T}_{c}\right)}^{-\frac{1}{2}}$ is the GL coherence length, ${T}_{c}= 7.7\,\text{ K}$ is the critical temperature. The fit shown in Fig. 1 by the dashed line is obtained with, ${B}_{1}=0.55 \,\mathrm{and}\, {B}_{2}=0.19$.

The phenomenological model presented by Arutyunov^8^ attributes the nMR effect observed in quasi-1D superconductors to quasiparticle charge imbalance which accompanies each phase slip event. The charge imbalance gives rise to a resistance

3 $${R}_{qp}={\rho }_{n}\left(\frac{2{\Lambda }_{Q}}{A}\right)\left({\tau }_{0}{\Gamma }_{PS}\right)$$

where ${\rho }_{n}$ is the normal state resistivity, ${\Lambda }_{Q}$ is the quasiparticle charge imbalance decay length, ${\Gamma }_{PS}$ is the average rate of the phase slips and ${\tau }_{0}$ is the duration of each phase slip event. The total resistance of the quasi-1D wire is given by the sum of ${R}_{qp}$ and the effective resistance ${R}_{ps}$= h $\frac{{\Gamma }_{PS}}{2eI}$ resulting from the phase changes associated with the phase slips. The charge imbalance decay length ${\Lambda }_{Q}$ decreases with the field according to

4 $${\Lambda }_{Q}^{H}={(\frac{4D{k}_{B}{T}_{c}{\tau }_{e}}{\pi \Delta })}^{1/2}{[1+\frac{3.52{k}_{B}{T}_{c}{\tau }_{e}}{\hslash {H}_{c}^{2}}{H}^{2}]}^{-1/4}$$

where $\mathrm{D}$ is the diffusion constant, ${\uptau }_{\mathrm{e}}$ the electron–phonon inelastic scattering time, and $\Delta$ is the energy gap. This expression is obtained by substituting ${\tau }_{S}^{H}$ in the expression for ${\tau }_{Q}$ in Arutyunov’s model (Eqs. 7 and 4, respectively, in Ref.^8^).The magnetoresistance behavior of a superconducting nanowire is, thus, governed by two competing processes: The rate of phase slips, ${\Gamma }_{PS}$, which increases with the magnetic field due to the suppression of $\Delta$, and charge imbalance decay length, ${\Lambda }_{Q}$, which decreases with the magnetic field due to decreases in the pair-braking time (${\tau }_{s}$). This competition dictates the behavior of the magnetoresistance: At low fields, the decrease in ${\Lambda }_{\mathrm{Q}}$ with the field dominates, giving rise to dR/dH < 0. At high fields, the increase in ${\Gamma }_{\mathrm{PS}}$ with the field dominates, giving rise to dR/dH > 0.

The effect of bias current on the charge imbalance relaxation length is qualitatively similar to that of a magnetic field

5 $${\Lambda }_{Q}^{I}={(\frac{4D{k}_{B}{T}_{c}{\tau }_{e}}{\pi \Delta })}^{1/2}{[1+\frac{D{\tau }_{e}}{{(3\sqrt{3}{I}_{C}\xi )}^{2}}{I}^{2}]}^{-1/4}$$

This expression is obtained by substituting ${\tau }_{S}^{H}$ in the expression for ${\tau }_{Q}$ in Arutyunov’s model (Eqs. 6 and 4, respectively, in Ref.^8^).The bias current decreases ${\Lambda }_{\mathrm{Q}}$, therefore, in measurements of R *vs.* I one may expect to observe a range of currents in which dR/dI < 0. However, as shown in Fig. 2, such a behavior has not been observed; the resistance monotonically increases with the current, indicating the domination of the phase slip contribution, ${R}_{ps}$, to the resistance throughout the entire current range. Indeed, contrary to magnetic field that slightly affects ${\Gamma }_{PS}$ due to variation of the gap, $\Delta$, with the field, the effect of bias current is much more pronounced as is apparent from Eq. (1). It is interesting to note that current suppresses the resistive transition anomaly observed in some superconducting nanowires^32–34^, which can be interpreted as inducing negative differential resistance. However, our wires show neither the resistive transition anomaly nor negative differential resistance.

The fast increase of ${\Gamma }_{PS}$ with current also explains the suppression of the nMR effect with current (Fig. 3b). In the competition between the positive magnetoresistance due to ${\Gamma }_{PS}$ and the negative contribution originating from ${\Lambda }_{\mathrm{Q}}$, the current tips the scales in favor of ${\Gamma }_{PS}$ and, as a result, the nMR is suppressed.

The effect of temperature is qualitatively similar to that of the current; as temperature increases, ${\Gamma }_{PS}$ increases and the positive magnetoresistance contribution to the total resistance increases. Since both temperature and current work in the same direction, namely increasing ${\Gamma }_{PS}$, it is clear that the effect of current is more pronounced at low temperature, and vice versa, the effect of temperature is more pronounced at low currents. As was mentioned above, Arutyunov’s model is applicable only near the transition temperature and provides two separate expressions for ${\Lambda }_{Q}$, as a function of magnetic field and current. Consequently, at this stage, a quantitative analysis of our results cannot be performed as our data includes results obtained far below T_c_ and in order to fit the model to our data we need an expression for ${\Lambda }_{Q}$ as a function of both field and current. We hope that our experimental work will inspire theoretical extension of Arutyunov’s model to low temperatures and to derive an expression for ${\Lambda }_{Q}$ that includes both field and current.

## Conclusions

The effect of transport current on the magnetoresistance can be qualitatively explained within the framework of the phenomenological model of Arutyunov, which attributes the magnetoresistance to the interplay between the resistance resulting from the rate of fluctuations via the Josephson relation, and an Ohmic contribution from the quasi-normal regions that accompany the fluctuation. Both temperature and current strongly increase the rate of fluctuations, thus suppress the nMR. Similar to the effect of the magnetic field, the current also reduces the quasiparticle charge imbalance length, thus one would expect to observe a non-monotonic behavior of the resistance not only with respect to magnetic field but also with respect to the current. However, the experiment shows no such a behavior; the resistance always increases monotonically with the current. The difference between the behavior with respect to the field and current can be attributed to the effect of the field on the fluctuation rate which occurs only through the dependence of the gap on the magnetic field, as opposed to the current which enhances more strongly the fluctuations rate. As the temperature and current qualitatively affect the nMR in a similar way, the temperature dependence of the nMR effect is more pronounced at low currents. Similarly, the current dependence of the nMR is more pronounced at low temperatures.

## Data Availability

The datasets used and analyzed during the current study are also available from the corresponding author on reasonable request.
